# Prognosis by Nuclear Units

**Published:** 1916-06

**Authors:** 


**Prognosis by Nuclear Units.** H. H. Seyl, Chicago, Ill. Med. Jour, May. Arneth showed long ago that the polymorphonuclear leucocytes may be classified by the number of nuclear lobes in each cell, which vary from 1 to 5. Normal blood shows 5, 35, 41, 17, 2% respectively. It has been the custom to estimate according to the percentage both ways from the dividing average by adding the first two columns and half of the total of the third column, counting the normal as 60% of the total number of polymorphous cells. Seyl holds that the proper way is to total the number of nuclear lobes. Normally, we have 5x1, 35x2, 41x3, 17x4, 2x5, a total of 276. Arrested cases of tuberculosis by this method gives a grand total of nuclear units of 215-250; incipient cases 195-215; moderately advanced cases 180-195; advanced cases 180 and downward. He cites a case of pernicious anaemia treated by splenectomy and transfusion (3 transfusions) in which the total increased from 113 to 151 two months later, with clinical improvement. A second case, same treatment, showed a decline 108-102 and ended fatally. A fatal case of lymphatic leukaemia gave a total of 100. Various other diverse cases apparently show that unless the index rises considerably above 100, death occurs.

